# IdeaDistiller—AI Support for Idea Synthesis in Concept Mapping: Algorithm Development and Validation Study

**DOI:** 10.2196/86877

**Published:** 2026-07-02

**Authors:** Chatrine Qwaider, Nora K Speicher, Anna E Genell, Mikael Holtenman, Lisa Vaughn, Frida Smith

**Affiliations:** 1 E-commons, Chalmers University of Technology, Chalmersplatsen 1, Gothenburg, 412 96, Sweden, 46 732501659; 2 Department of Natural Language Processing, Mohamed bin Zayed University of Artificial Intelligence, Abu Dhabi, United Arab Emirates; 3 Regional Cancer Centre West, Gothenburg, Sweden; 4 Cincinnati Children’s Hospital Medical Center, University of Cincinnati College of Medicine Department of Pediatrics, Cincinnati, OH, United States; 5 Educational and Community-Based Action Research, University of Cincinnati College of Education, Criminal Justice, and Human Services, Cincinnati, OH, United States; 6 Department of Technology Management and Economics, Chalmers University of Technology, Gothenburg, Sweden

**Keywords:** concept mapping, semantic clustering, topic modeling, bidirectional encoder representations topic modeling, BERTopic, human-in-the-loop, qualitative research automation

## Abstract

**Background:**

Concept mapping (CM) is a widely used mixed method research approach for structuring and visualizing complex ideas across various fields, such as the health sciences. A critical bottleneck in the CM process is the idea synthesis phase, which remains labor-intensive, subjective, and consequently challenging to scale for large datasets.

**Objective:**

In this study, we propose IdeaDistiller, a semiautomated solution based on semantic clustering to optimize the idea synthesis step while maintaining methodological rigor through a human-in-the-loop approach.

**Methods:**

Using 9 health care–related datasets in English and Swedish, we systematically evaluated different embedding models, dimensionality reduction techniques, and clustering algorithms to identify robust and reproducible parameter settings for the proposed approach. IdeaDistiller clusters participant-generated ideas based on semantic similarity to identify similar ideas with different wording, suggests representative and unique ideas per cluster, and provides coherence scores and sorted outputs to aid manual validation.

**Results:**

Our findings suggest that IdeaDistiller may substantially reduce the manual effort involved in idea synthesis while preserving quality and transparency. However, human expertise remains indispensable for validating and refining cluster outputs.

**Conclusions:**

Integrating semiautomated methods into the CM workflow offers significant potential for improving the efficiency, scalability, and rigor of the CM process. Building on our work will enable the exploration of larger multilingual datasets and integration into future CM studies.

## Introduction

### Background

Concept mapping (CM) is a structured research method designed to visualize and organize thoughts, ideas, or knowledge concerning a specific topic by creating graphical representations of concepts and their interrelationships [1]. Widely used in disciplines such as education, psychology, health sciences, and other social sciences, CM is an integrative mixed methods research methodology for exploring and analyzing complex conceptual domains. It includes both qualitative and quantitative data collection and analysis in sequential steps, where each step builds on the previous one, as described below [2]. One of the strengths of this method is the active involvement of stakeholders throughout the process. Depending on the project and the research question, the participants can be more or less involved in all stages described in the following sections [3].

### CM Methodology

**Figure 1.**
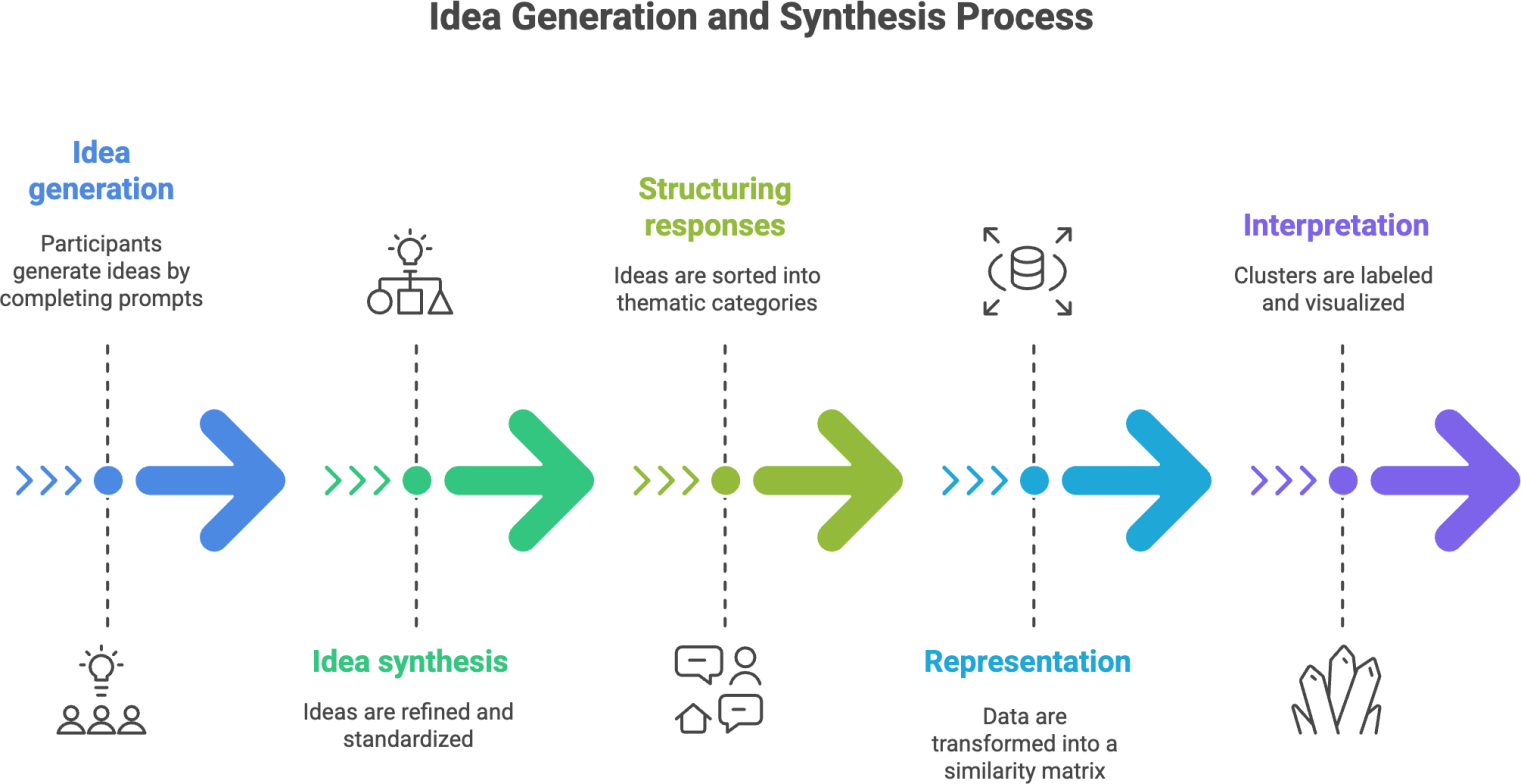
Process of concept mapping methodology.

After the initial preparation stage, the CM methodology typically comprises the following sequential stages, as shown in Figure 1. Normally, idea synthesis is part of idea generation, but for clarity in this study, it is defined here as an additional step in the process.

#### Idea Generation

The research team formulates a focused research question, typically structured as an incomplete sentence or prompt. Participants individually complete this sentence, usually generating between 1 and 5 responses, referred to as ideas. Given the volume of data collected, a subsequent idea synthesis process is necessary to manage and refine the input so that the ideas are manageable for the sorting step.

#### Idea Synthesis

Idea synthesis involves a series of refinement steps to produce a concise yet representative set of ideas for further analysis, referred to as the set of unique ideas for the study. This process includes eliminating identical or highly similar ideas, preserving unique and relevant contributions, removing off-topic ideas, and segmenting complex or compound ideas into more granular units. Additionally, grammatical and typographical corrections are applied to standardize the text. The overarching goal is to reduce the initial set of ideas to a manageable number, typically no more than 100 distinct ideas, while preserving the conceptual breadth and diversity of the original input [1,4].

#### Structuring the Ideas

A subset of participants individually sorts the unique ideas into thematic categories, ensuring that each idea is placed in only 1 category. This process can be done manually or, as in most cases, using designated card-sorting software. Participants also assign descriptive labels to the categories they create.

#### Representation

The sorting data are transformed into a similarity matrix that captures how often pairs of ideas are grouped. Multidimensional scaling is then applied to represent these similarities or dissimilarities on a 2D plane. The multidimensional scaling output is subjected to hierarchical cluster analysis to develop a hierarchy of clusters. The research team reviews successive cluster solutions to determine the optimal number of clusters, selecting the most meaningful and interpretable configuration while balancing sufficient yet manageable detail [5]. Depending on the project, participants can also be part of this stage, as well as in the final step, interpretation.

#### Interpretation

Clusters are labeled based on the category names suggested during sorting. The final concept map visually presents the ideas’ spatial coordinates and delineated clusters, offering a structured and interpretable visualization of the conceptual domains.

The principal objective of CM is to provide a clear, visual representation of a conceptual space and set of ideas, thereby enhancing the understanding of underlying structures and relationships among the ideas. A defining characteristic of CM is the active engagement of stakeholders at multiple stages of the research process, including data collection, analysis, and interpretation, making it highly compatible with participatory research methodologies [3].

Despite its structured framework, CM poses a number of challenges. Criticisms, even from the originators, include the potential for subjectivity and bias, particularly during qualitative phases, as well as the method’s high demands on time and resources for researchers and participants [6]. The idea synthesis phase is particularly concerning, as it involves refining, consolidating, and preparing ideas for subsequent sorting and analysis [4].

While the core CM methodology is well-established, the growth of large-scale, digitally mediated data collections necessitates more efficient, rigorous, and partially automated approaches to the idea synthesis step.

### Related Work

The idea synthesis phase in CM has traditionally relied on manual, labor-intensive procedures conducted by research teams through iterative consensus-building exercises. A range of approaches has been documented in the literature. For instance, Ashe et al [7] describe a multistage process in which individual review of items was followed by several collaborative meetings to finalize the list of unique ideas. Similarly, Windsor [8] employed 5 independent coders to identify redundancies, after which consensus was reached on the items to retain. Hassmiller Lich et al [9] introduced a more advanced strategy by comparing an initial set of 830 ideas with a final curated set of 97 to assess representativeness. Pantha et al [2] conducted a review indicating that while approximately one-third of studies provided an explicit rationale for their sample size, three-quarters described their idea synthesis process in detail. However, concerns about methodological transparency remain. For example, McLinden [10] criticized the often insufficient reporting of synthesis procedures, arguing that such omissions hinder the ability to evaluate the representativeness and validity of the final idea sets. These studies underscore the variability in practice and the need for more standardized and transparent approaches to idea synthesis in CM research.

Despite growing awareness of the importance of rigorous idea synthesis, inconsistencies persist in methodological reporting and implementation. Even though digital technologies have enabled larger-scale data collection, they have also further compounded the challenges and increased the burden on researchers to conduct transparent and methodologically sound synthesis. While Kane and Trochim [1] emphasized that CM relies on a combination of technological support and human qualitative judgment—a “high-tech, high-touch” approach—there is a growing need for methods that blend automation with interpretive rigor without compromising the qualitative depth of the process.

To address all the aforementioned challenges, this study presents the development and evaluation of IdeaDistiller, a semiautomated tool designed to support the idea synthesis phase of CM. By leveraging machine learning techniques, our approach aims to reduce researchers’ burden, enhance methodological transparency, and preserve qualitative depth. We demonstrate the tool’s utility through a series of exploratory benchmarks across diverse English and Swedish datasets, providing a framework for more efficient and reproducible conceptual analysis in large-scale studies.

## Methods

### Design Rationale and Requirements

Recognizing that idea synthesis is inherently a qualitative task requiring nuanced judgment, we adopted a human-in-the-loop (HITL) approach, integrating automated techniques with expert oversight. This strategy aligns with the recommendation by Kane and Trochim [1] that, while technology can support the CM process, human interpretive engagement remains essential. We acknowledge that the distillation of ideas is not a discovery of an objective “ground truth” but rather a subjective interpretive process in which granularity and categorization depend on the researcher’s perspective. Overall, we aimed at developing a tool that fulfills the following requirements:

- Redundancy reduction: identifying and eliminating duplicate or semantically similar ideas to minimize repetition within the idea set.
- Semantic alignment: assessing and organizing ideas by measuring their semantic similarity to each other.
- Preservation of unique contributions: safeguarding novel and valuable ideas to maintain the richness and diversity of participants’ input.

Our approach is based on the principle that all ideas can be clustered into semantically coherent groups, where each group consists of ideas conveying the same or a similar idea in different formulations, and the main idea for each group is distinct from all other groups. Creating such a clustering enables the researcher to subsequently extract one representative idea from each group, ensuring that all extracted ideas collectively represent the diversity of the complete dataset.

We explored several approaches to clustering ideas based on their semantic similarity. In the following sections, we describe the data, clustering methods, and evaluation metrics used, as well as the output created to facilitate the easy integration of human feedback.

### Datasets

This study used data from various independent CM studies. Each dataset comprised two components: (1) the complete set of raw ideas provided by participants and (2) the manually identified set of unique ideas, which served as the reference for evaluating the performance of the computational methods developed.

In total, 9 datasets were analyzed: 6 in English and 3 Swedish datasets. The English datasets cover diverse topics within the health and health care domains, including obesity, stress, medical services [11], drug abuse [12], and aspects of suicide screening [13]. The datasets covering the first 3 topics originally contained Spanish ideas collected from Latino participants in Cincinnati; however, their English translations were used in this study. The Swedish datasets addressed cancer rehabilitation [14], the support structures available to patients and their families (the “Kraftens hus” initiative) [15], and the use of development plans as a tool in patient care [16].

Before clustering, exact duplicate entries, which were particularly frequent among shorter ideas, were removed as they would not contribute additional unique ideas. However, within our approach, it is possible to retain duplicates to allow for a quantitative follow-up analysis.

Table 1 provides an overview of the datasets, along with their sizes and the number of manually identified unique ideas. The datasets vary in thematic focus, language, participant demographics, and dataset size, providing a robust basis for evaluating the generalizability and performance of the proposed computational approach.

**Table 1.** Datasets used in the study.

Dataset	Language	Original ideas^a^	Unique ideas
Obesity	English	406	100
Stress	English	670	97
Medical services	English	697	96
Drug abuse	English	162	75
Results of suicide screening	English	462	80
Important parts of suicide screening	English	415	80
Cancer rehabilitation	Swedish	525	67
Cancer support center	Swedish	121	72
Development plans	Swedish	375	100

a Number of original ideas after the removal of duplicates.

### Semantic Clustering

Semantic similarity identifies latent patterns within textual datasets by uncovering recurring groups of words that represent underlying themes or topics. Traditional methods, such as latent Dirichlet allocation [17,18], primarily rely on word frequency statistics to discover topics, which makes them limited in their ability to find semantic similarity in sentences with very different wording. Therefore, our approach builds on the BERTopic (bidirectional encoder representations topic modeling) framework [19-21], a technique that incorporates textual relationships and semantic meaning.

BERTopic follows a flexible pipeline comprising several steps. Since our primary interest lies in grouping each idea into a semantically coherent cluster, we focus on the first three stages of the pipeline: (1) embedding the ideas into a dense vector space, (2) reducing the dimensionality of the embedding space, and (3) clustering the embedded original ideas to form semantically related groups.

The core principle of BERTopic is embedding sentences into a high-dimensional embedding space to transform textual data into dense vector representations before clustering them. Semantic similarities between texts are reflected by their proximity in the vector space. These embeddings allow for a more nuanced understanding of similarity beyond simple word counts. In this study, we used several different pretrained embedding models, adapted to different languages, to create the sentence embeddings.

Through this process, all ideas are assigned to clusters, each containing original ideas with similar semantic meanings. CM researchers can then extract one or more representative ideas from each cluster. Each of the 3 mentioned steps offers multiple methodological choices, and we selected the specific methods that showed the most robust results across our exploratory study. While this process provides a good starting point, manual refinement is still needed. To simplify this task for the researcher, we additionally sort all ideas based on their cluster assignment and the semantic similarity between the clusters; that is, we organize the clusters such that those covering similar themes are positioned closer together in the output. To achieve this, we applied hierarchical clustering to the topic representations and used the resulting dendrogram to order the output.

### Evaluation Metrics

Standard evaluation metrics for topic modeling, such as coherence scores and perplexity, assess the semantic consistency and interpretability of the identified topics. In our case, however, the availability of manually curated unique ideas from previous CM studies enabled more targeted evaluation measures. Rather than measuring “correctness” in an absolute sense, these metrics quantify the degree of alignment between the computational output and a specific, human-led reference synthesis. To quantify the performance of IdeaDistiller, we developed 2 coverage-based metrics that assess the alignment between the computationally generated clusters (without human refinement) and the expert-identified unique ideas. These metrics are based on the heuristic that an optimal alignment with the reference standard would produce as many clusters as there are unique ideas, with 1 unique idea representing each cluster. Therefore, the metrics used consider the overlap between computational clustering and curated unique ideas, which is the number of clusters containing at least 1 unique idea. The 2 metrics are defined as follows:


$$\text{Coverage of clusters to unique ideas}=\frac{\text{overlap}}{n\_unique\_ideas}$$

where n_unique_ideas represents the total number of manually curated unique ideas. This metric evaluates the proportion of unique ideas represented by the computational clusters. In other words, this metric tells us how many of the unique ideas we would obtain if we extracted exactly 1 unique idea from each cluster and how many this computational approach would miss:


$$\text{Proportion of clusters with unique ideas}=\frac{\text{overlap}}{n\_clusters}$$

where n_clusters is the total number of computationally generated clusters. This metric assesses the proportion of clusters containing at least 1 manually identified unique idea, highlighting the extent to which the computational clusters align with expert curation. A value below 1 indicates clusters without a unique idea, which can be either due to suboptimal clustering (ie, the additional clusters do not consist of new ideas but should be a part of another cluster containing a unique idea) or due to the clustering identifying new aspects that the human evaluator has not considered.

As an example, consider a scenario with 100 clusters and 96 unique ideas, where 62 clusters contain at least 1 unique idea. In this case, the 2 metrics are calculated as follows:


$$\text{Proportion of Clusters with Unique Ideas}=\frac{62}{100}=0.62$$

$$\text{Coverage of Clusters to Unique Ideas}=\frac{62}{96}\approx 0.65$$

This example indicates that 38% (38/100) of clusters do not contain a manually curated unique idea, while 35.41% (34/96) of unique ideas are not captured when extracting 1 idea per cluster. Together, these metrics provide a comprehensive assessment of the representativeness and completeness of the clustering results. It is important to note that a lack of perfect correspondence (values below 1) does not necessarily indicate “error” by the model. Because unique ideas in CM are interpretive and dependent on the researcher’s chosen level of granularity, discrepancies may represent valid alternative structures. Therefore, these metrics should be interpreted as a measure of how closely the IdeaDistiller mimics the specific synthesis decisions made by previous expert researchers.

While these 2 metrics aim to evaluate the results from 2 different perspectives, we applied the pipeline by choosing the number of clusters to be equal to the number of manually curated unique ideas, which resulted in identical values for both metrics. Therefore, in the following, we will report only one of the metrics.

### Topic Coherence

Although coherence was not used to select the best-performing model, we computed a coherence score for each idea to support researchers during the manual evaluation phase. In our setting, coherence measures the degree of semantic similarity among all ideas within the same cluster, with higher coherence indicating greater internal consistency. This score can help assess how well an idea fits within its assigned cluster, allowing for special focus on ideas with low coherence scores during the postcomputational or refinement phase. In practice, we computed embeddings for all individual ideas using a pretrained sentence-BERT (bidirectional encoder representations from transformers) model [22]. The coherence score of each idea is then calculated as the cosine similarity between its embedding and the mean embedding of all ideas in its assigned cluster.

### Generated Output

Our approach, IdeaDistiller, produces an output file designed to support CM researchers during the idea synthesis phase. This file includes a comprehensive table listing all participant ideas alongside the results of the computational analysis, facilitating manual review and refinement.

The output contains the following components:

- Cluster labels: each idea is accompanied by an assigned cluster label, indicating the group to which the idea belongs based on semantic similarity.
- Sorting: to aid manual evaluation, the ideas are sorted according to their cluster assignments (ie, similar ideas should appear close to each other in the file), and clusters are organized based on semantic similarity. This structure enables researchers to easily assess whether adjacent clusters are sufficiently similar to warrant merging, potentially representing them with a common, unique idea.
- Suggested unique ideas: the file provides a computationally suggested representative idea for each cluster. When available, original, manually curated, and unique ideas are also included for comparison. This feature is useful when applying the tool to previously filtered datasets or when evaluating the alignment between manual judgments and computational outputs.
- Coherence scores: semantic coherence scores are provided for each idea, offering insights into the internal consistency and quality of the clustering process. A low coherence score for an idea may indicate a poor fit within its assigned cluster and can prompt further review and adjustment.

These outputs offer researchers a structured foundation for conducting a comprehensive evaluation and refinement of the results to finalize the idea synthesis process.

### Ethical Considerations

All projects that contributed data for this work have been ethically assessed and/or approved. Further details can be found in the respective manuscripts [11-16]. For this study, permission to use the data was granted by the data collectors, and no actual outcomes are reported. The data were used exclusively for the development and testing of the tool and are neither required for its further use nor can they be retrieved from the tool.

## Results

As outlined in the *Datasets* section, the datasets used vary in characteristics such as size, topic, language, and participant demographics. This diversity allows us to evaluate the usefulness and generalizability of the proposed approach under diverse conditions.

### Experiments and Parameter Selection

#### Sensitivity Analysis and Parameter Exploration

Given that a definitive “ground truth” for idea synthesis is rarely available in real-world research contexts, our experimental grid is intended as a sensitivity analysis. We aimed to demonstrate the robustness of the IdeaDistiller framework across a wide range of configurations, rather than to nominate a single “optimal” model for all future applications. We aimed to maintain a one-to-one correspondence between clusters and unique ideas and selected the number of clusters to match the number of unique ideas in each dataset using the metrics described above.

Using BERTopic, we explored different parameter configurations for the three main components of the computational pipeline: (1) embedding models, (2) dimensionality reduction methods, and (3) clustering algorithms.

The specific parameters and methods tested are summarized in Table 2. The total number of experiments conducted per dataset is as follows:


Total experiments=(Number of clustering algorithms)×(Number of embedding models)×(Number of dimensionality reduction methods×Number of dimensions)

Accordingly, the datasets for the experiment are as follows:

- For the English datasets: 3×3×(2×9)=162 experiments per dataset
- For the Swedish datasets: 3×2×(2×9)=108 experiments per dataset

This allowed us to assess the impact of different parameters and evaluate performance under diverse configurations. Our results showed that no single parameter configuration consistently yielded the best performance across all datasets, reflecting the heterogeneity of the datasets.

**Table 2.** Overview of the different parameter settings that were tested.

Parameters	Options
Clustering	K-means clustering, agglomerative clustering, and spectral clustering.
Embedding	English: fastText^a^, SentenceTransformer, Flair^b^ Swedish: KB-BERT^c^, SentenceTransformer
Dimensionality reduction	Truncated SVD^d^, PCA^e^ (with dimensions varied from n=2 to n=10)

a fastText: Facebook artificial intelligence word embedding model.

b Flair: natural language processing embedding framework.

c KB-BERT: Kungliga Biblioteket bidirectional encoder representations from transformers.

d SVD: singular value decomposition.

e PCA: principal component analysis.

#### Clustering Algorithm Selection

We evaluated multiple clustering algorithms to determine the most suitable method for our approach. We explored hierarchical density–based spatial clustering of applications with noise due to its ability to discover clusters of varying densities without requiring a predefined number of clusters. However, initial trials resulted in very high numbers of clusters and outliers, reflecting the complexity of the dataset. We, therefore, concluded that the absence of explicit control over the number of clusters rendered hierarchical density–based spatial clustering of applications with noise less appropriate for our application.

Overall, we compared the performance of 3 clustering algorithms across all datasets: k-means, agglomerative clustering (with Ward linkage), and spectral clustering.

**Figure 2.**
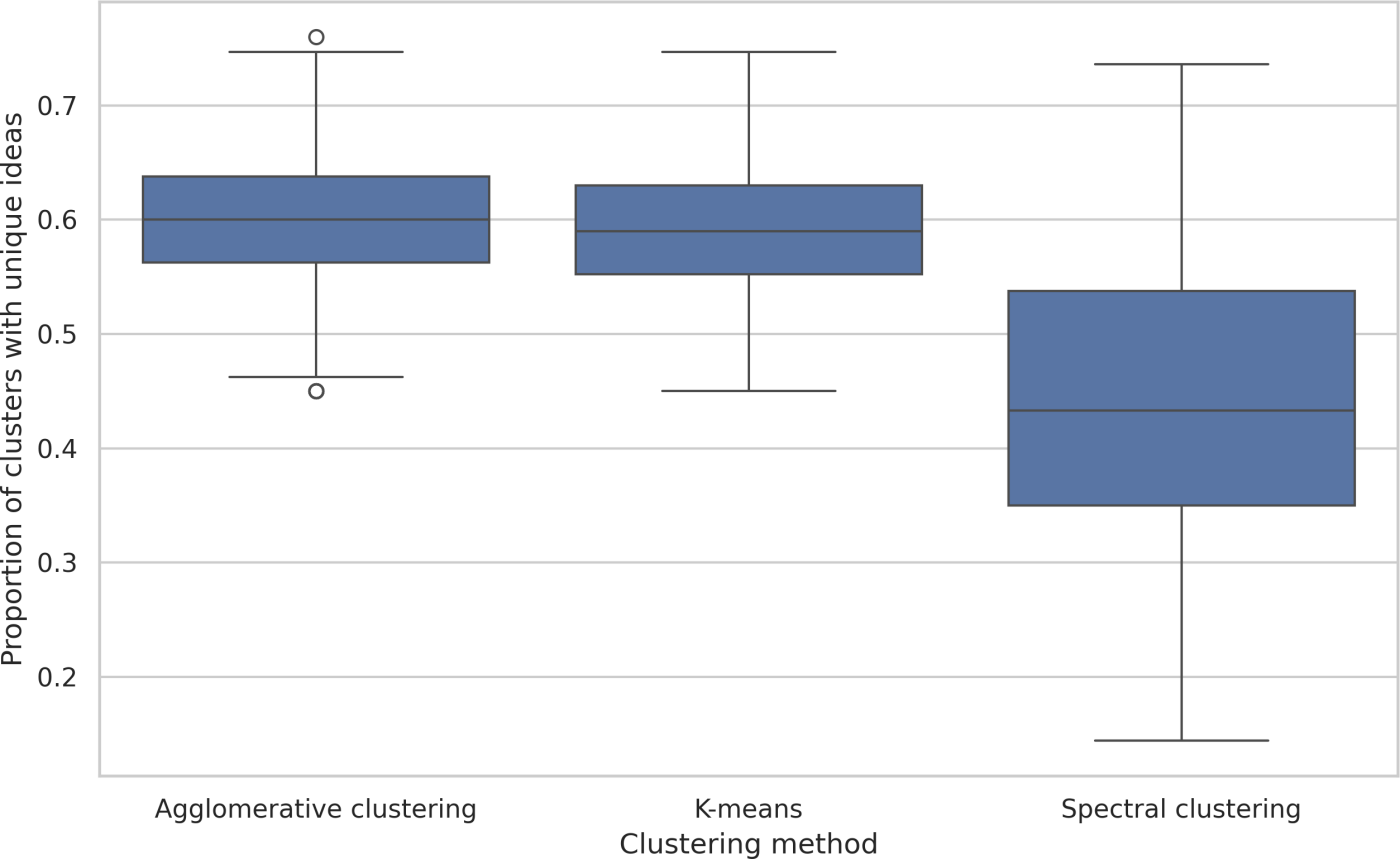
Box plots of the 3 clustering algorithms (k-means, agglomerative clustering, and spectral clustering) showing their proportions of clusters with unique ideas.

As illustrated in Figure 2, spectral clustering appeared to show less stable performance and tended, on average, to yield lower results compared with the other methods. In contrast, k-means and agglomerative clustering seemed to perform at broadly similar levels across the experiments. These impressions are based on visual inspection of the box plots and should, therefore, be interpreted with appropriate caution.

We suggest agglomerative clustering as a practical baseline configuration for the pipeline. While k-means is a robust alternative, its sensitivity to random initialization can yield varying outcomes across different runs unless specific measures—such as fixed seeds or multiple restarts—are implemented. We prioritized agglomerative clustering primarily for its inherent determinism, as well as its established use within the field, contributing to its acceptance among researchers. In the following sections, we report the performance based on agglomerative clustering only, excluding the other clustering approaches.

#### Embedding Models

Embedding models are used to represent sentences as numerical vectors within a high-dimensional space, where the proximity between vectors captures semantic similarity. Various embedding models exist, often tailored to specific languages, which can significantly influence clustering performance.

For the English datasets, we evaluated 3 models: fastText, SentenceTransformer (all-MiniLM-L6-v2), and Flair. For the Swedish datasets, we tested KB-BERT (Kungliga Biblioteket bidirectional encoder representations from transformers; bert-base-swedish-cased) and SentenceTransformer (paraphrase-multilingual-MiniLM-L12-v2).

**Figure 3.**
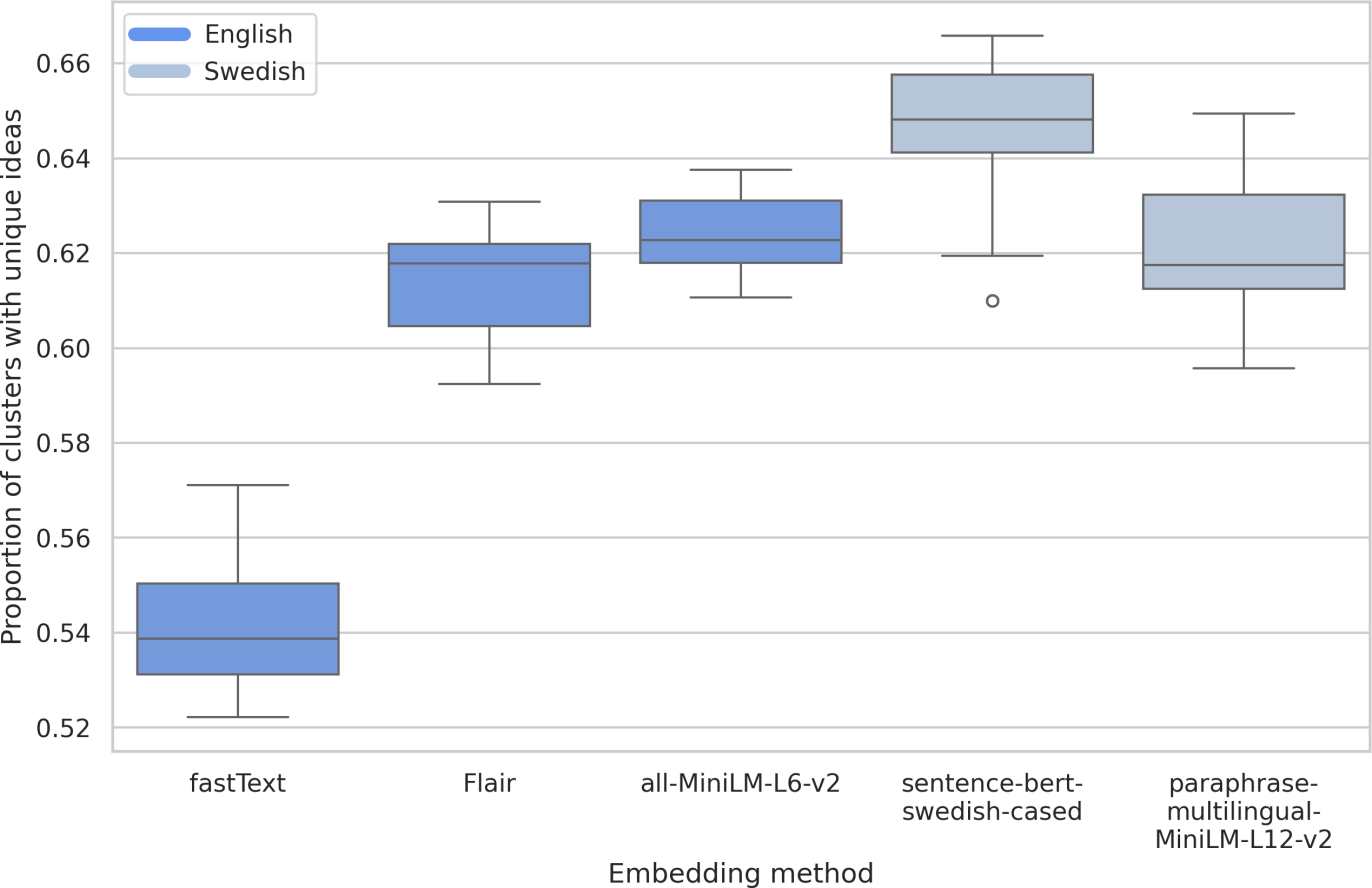
Box plots of embedding models showing their proportion of clusters with unique ideas using agglomerative clustering on the English and Swedish languages. fastText: Facebook artificial intelligence word embedding model; Flair: natural language processing embedding framework; MiniLM: miniature language model.

Our experiments demonstrated that, for the available English datasets, the SentenceTransformer (all-MiniLM-L6-v2) showed the highest mean performance within this exploratory study, as shown in Figure 3, yielding better alignment with the manually curated unique ideas. For our Swedish datasets, KB-BERT (bert-base-swedish-cased) showed the highest performance; however, given the small number of Swedish datasets, these results should be interpreted as indicative rather than definitive (Figure 3). Nevertheless, we selected KB-BERT because its training on Swedish-specific corpora is expected to improve its ability to capture linguistic nuances unique to the language.

In the following sections, we report the performance of the results based on agglomerative clustering combined with the selected embedding models.

#### Dimensionality Reduction

Dimensionality reduction is applied to high-dimensional embeddings to reduce computational complexity while preserving the most relevant semantic information. Different techniques emphasize the preservation of distinct aspects of the original data structure, which has an impact on downstream clustering performance.

Although uniform manifold approximation and projection is the default technique in BERTopic and often yields high-quality embeddings, we excluded it from our experiments. While stochastic methods like uniform manifold approximation and projection can be made reproducible through fixed random seeds, we opted for deterministic alternatives (truncated singular value decomposition [SVD] and principal component analysis) to ensure consistent results across different computational environments and versions. This choice was made to prioritize practical deployment and ease of use, and does not imply that nondeterministic methods are theoretically inferior for this task.

**Figure 4.**
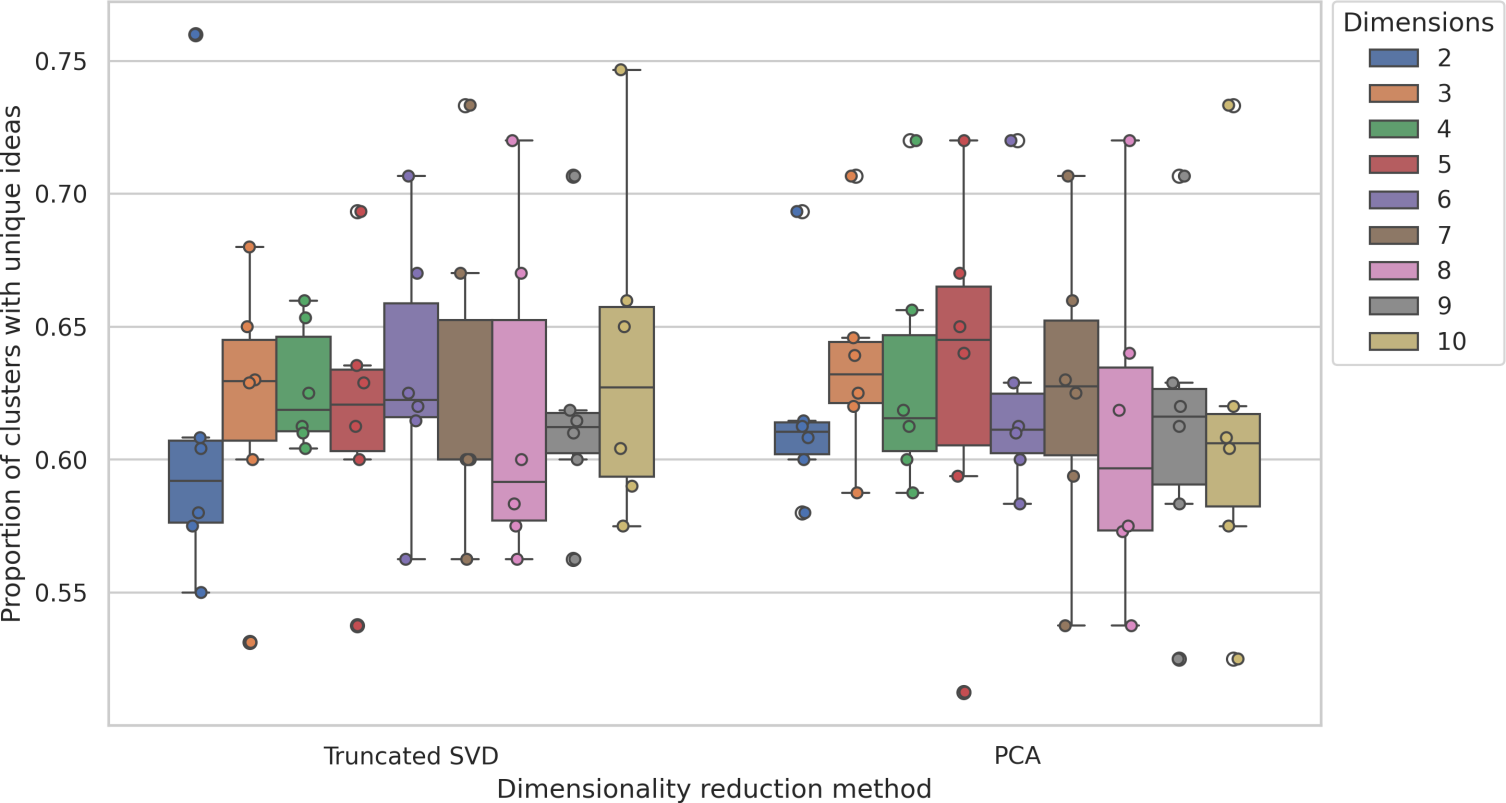
Box plots of dimensionality reduction methods (truncated SVD and PCA) with varying numbers of dimensions, showing their proportion of clusters with unique ideas for the English dataset. PCA: principal component analysis; SVD: singular value decomposition.

**Figure 5.**
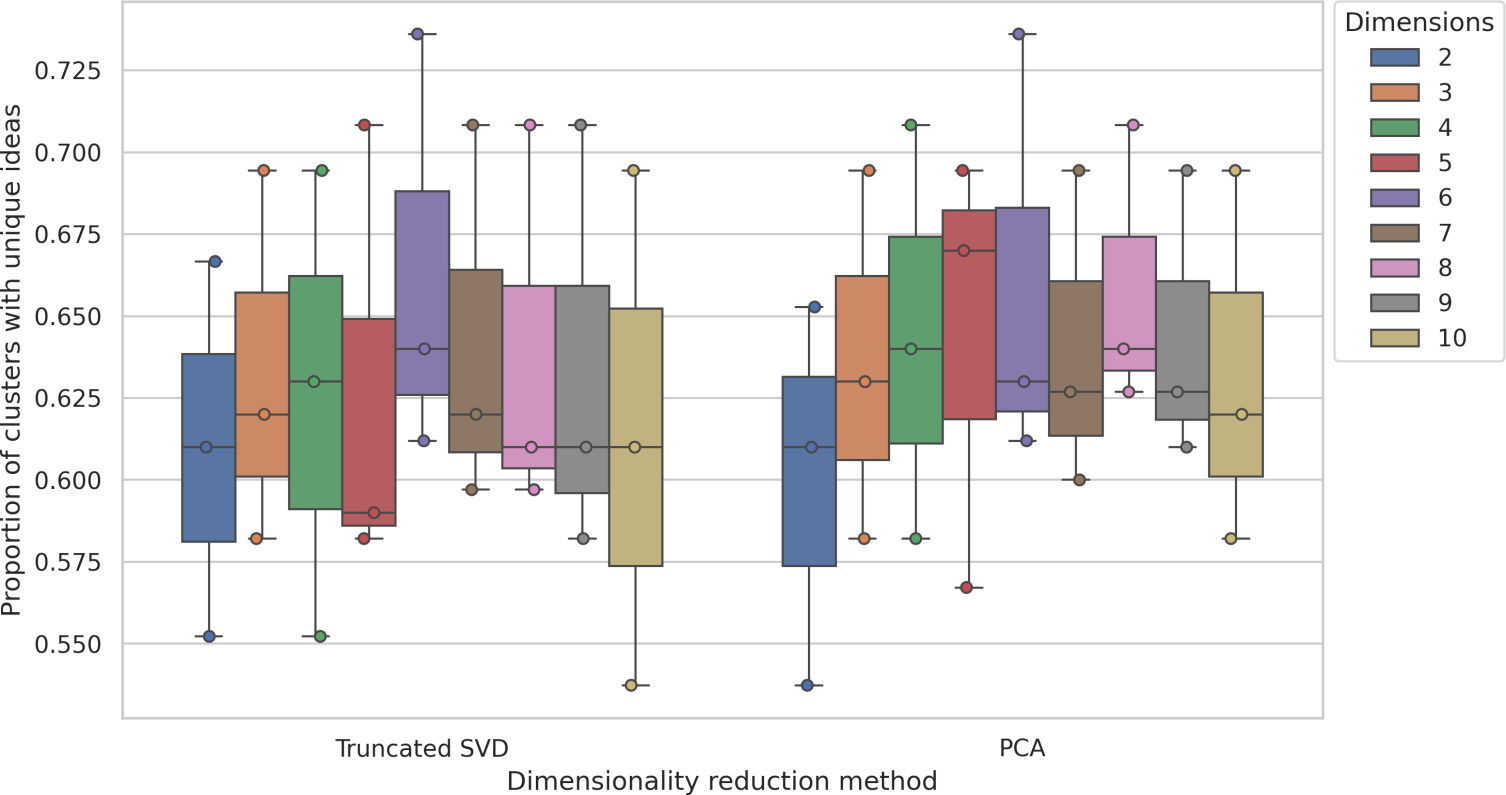
Box plots of dimensionality reduction methods (truncated SVD and PCA) with varying numbers of dimensions, showing their proportion of clusters with unique ideas for the Swedish dataset. PCA: principal component analysis; SVD: singular value decomposition.

**Figure 6.**
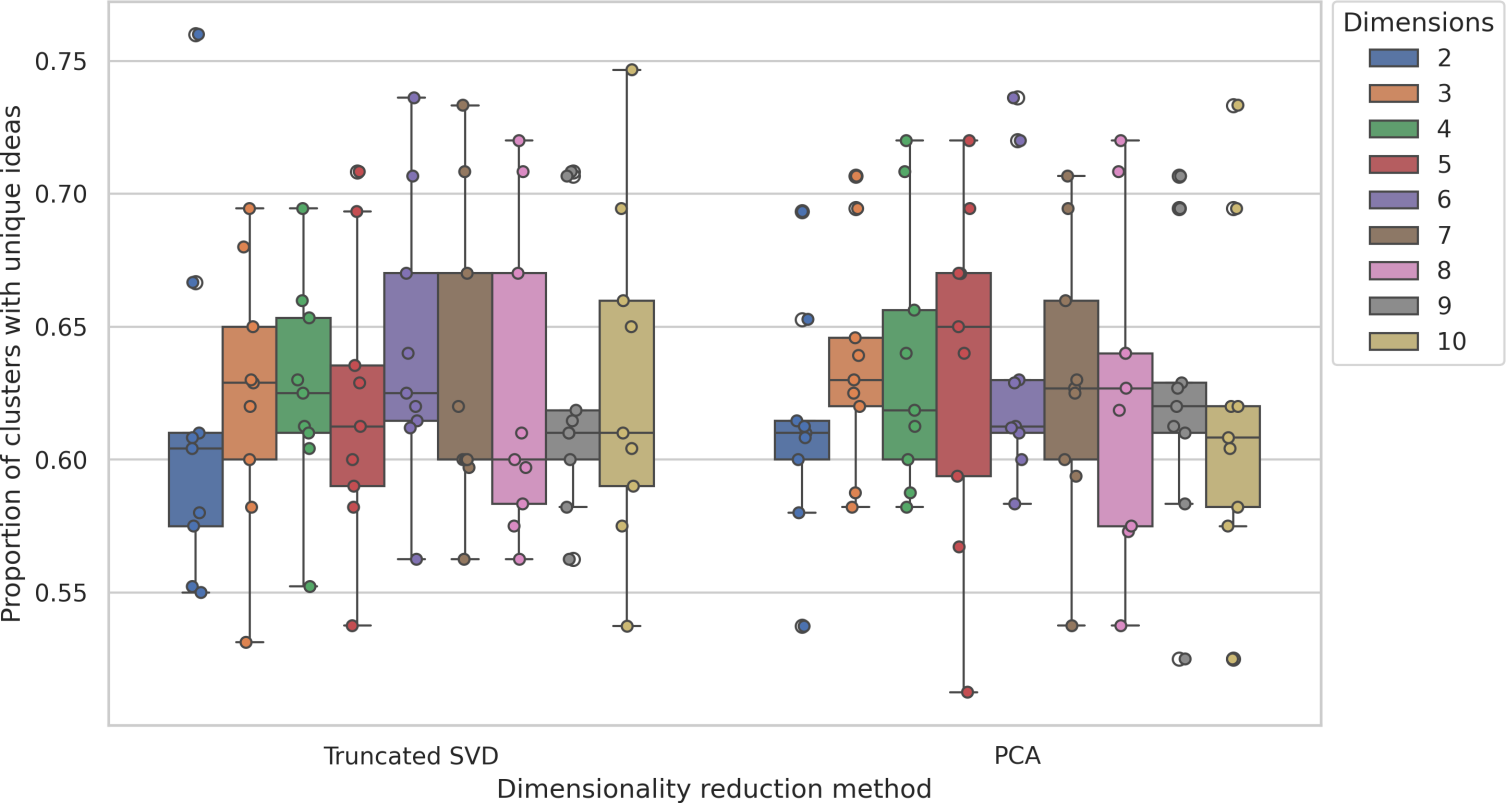
Box plots of dimensionality reduction methods (truncated SVD and PCA) with varying numbers of dimensions, showing their proportion of clusters with unique ideas for both languages. PCA: principal component analysis; SVD: singular value decomposition.

Each method was evaluated across a range of dimensions (from n=2 to n=10), with results summarized in Figures 4 to 6. Language-specific analyses revealed that for our English datasets, the best performance seemed to be achieved by using truncated SVD with a higher dimensionality (n*=*10), whereas for the available Swedish datasets, truncated SVD with a lower dimensionality (n=6) showed, on average, slightly better results. However, given the small number of datasets analyzed (6 in English and 3 in Swedish) and the associated variability in the results, we recommend using truncated SVD with a lower dimensionality (n=6) across both languages. This setting seems to provide more robust and stable performance across all the datasets, whereas a higher dimensionality (n=10) exhibited greater variance in clustering quality when considering both English and Swedish datasets.

With these selected methods, the average performance of IdeaDistiller is 0.63 for English datasets and 0.67 for Swedish datasets, meaning that 63% (n=100) and 67% (n=100) of clusters, respectively, contain at least 1 manually curated unique idea, while 37% (33% for Swedish) of the clusters do not contain a unique idea. The latter can be due to suboptimal cluster structure; that is, those clusters could be merged with other existing clusters; however, in some cases, these clusters might provide new, additional perspectives on the topic. These results indicate an existing correlation between manual and computational results, meaning that IdeaDistiller can support the researcher in their analysis. At the same time, they also showcase that the output of IdeaDistiller will need human revision to ensure methodological rigor. The clustered and sorted lists of original ideas produced by IdeaDistiller therefore represent an intermediate step in the idea synthesis process. While this tool does not provide a final solution for idea synthesis, it offers structural assistance and reduces cognitive load on the researchers.

#### Number of Clusters

**Figure 7.**
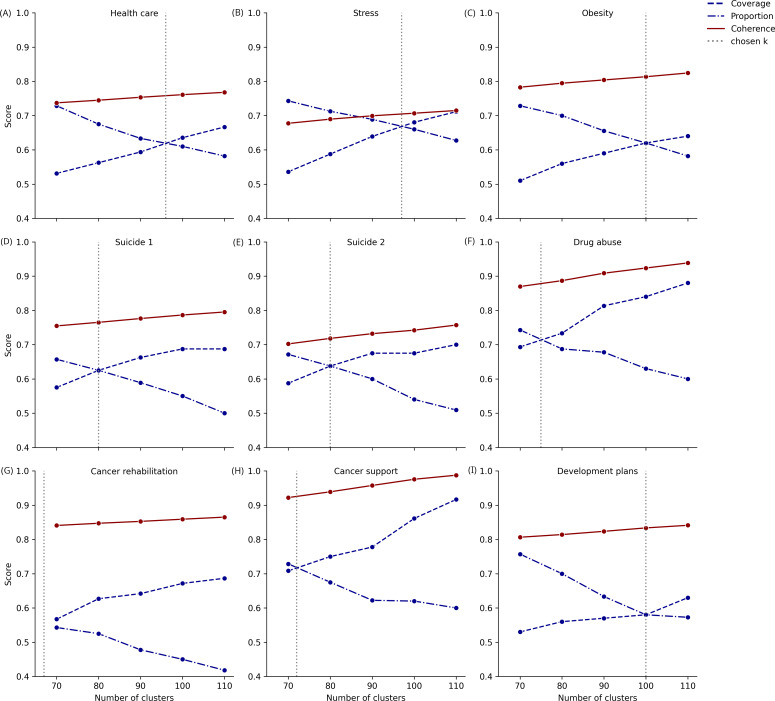
Impact of cluster number (*k*) on proportion, coverage, and coherence scores across 9 datasets.

In our previous experiments, the number of clusters, *k,* was always set to the number of manually curated unique ideas to evaluate how well IdeaDistiller reproduces these results. With a new CM study, however, this value will not be available, but the researcher might instead use a few standard values. To investigate the effect of different cluster numbers on the results, we varied *k* between 70 and 110 while keeping the suggested options for all other parameters. The results were evaluated using both the proportion of clusters with unique ideas (called proportion) and the coverage of clusters to unique ideas (called coverage), as well as the coherence score, which is independent of the manually curated unique ideas. Figure 7 shows that, due to the way they were constructed, proportion and coverage have an inverse relationship, crossing at the true value of manually curated ideas. The effect of a change in *k* depends on the dataset. Within the considered range of *k*, the change in the proportion score lies in the range of 0.12 to 0.18, while the coverage score shows slightly higher differences, ranging from 0.1 to 0.21 across our datasets. The average coherence score for each dataset monotonically increases with the number of clusters. This is expected, since it measures how closely connected the ideas within a cluster are, with more clusters allowing for better separation of the different aspects covered by the dataset. Based on these results, we generally suggest choosing *k* slightly higher than the number of unique ideas one is aiming for. This way, the user obtains more coherent clusters. At the same time, the clusters become smaller and possibly overlapping in content. To address this, IdeaDistiller provides an output file where the clusters are sorted semantically, such that similar clusters are grouped together and can easily be identified when reviewing the list.

### Coherence Calculation

To provide a concrete example of how coherence scores reflect the semantic unity of the output, Table 3 illustrates a cluster (ID 86) from the English dataset “Stress.” The scores represent the cosine similarity between individual statements and the cluster centroid. Higher scores (eg, 0.76) correspond to statements that align closely with the core topic, while lower scores (eg, 0.52) indicate less closely related concepts like “support.” This allows researchers to quickly validate the thematic consistency of the tool’s output and identify potential outliers.

**Table 3.** Representative clusters and associated coherence scores, illustrating semantic proximity to the cluster centroid.

Idea	Coherence score
Look for more information	0.76
Have more information	0.70
More information	0.60
More support	0.52

### Illustrative Case Study

We conducted a small audit on one of the English datasets (“Drug abuse”) to gain insights into the semantic clusters without unique ideas. We found that, in many cases, these clusters did not contain novel content but slight variations of already selected ideas. However, some of these clusters contained ideas that could be deemed “novel” depending on the focus of the study. One example is 3 statements on background checks (“give background checks when you apply for a license,” “background checks on patients,” and “ask for consensual background checks to know if a person is on drugs or has experienced…”), with none of them being included in the list of unique ideas. For the given study, these ideas were considered out of scope and at the same time loosely related to 2 retained unique ideas (“The federal government should run a program that takes convicted drug dealers/users into mandatory rehab” and “Hold addicts accountable for staying clean and away from drugs”). However, depending on the focus of the study, an idea specifically about background checks could be a useful addition. This example illustrates how scope and granularity decisions shape what is included as a unique idea and shows that the tool provides a valuable starting point for idea synthesis. However, it remains independent of these study-specific decisions and therefore does not replace researcher judgment.

## Discussion

### Principal Findings

This study shows that semantic clustering can be used to support the idea synthesis process in a CM study. For 9 different datasets, IdeaDistiller was able to reproduce expert synthesis with an average alignment of 64%, highlighting both the value of the tool in organizing the original ideas as well as the need for expert involvement in the curation process. The latter is facilitated by providing sorted lists of all original ideas, based on which the results can easily be adjusted.

Topic modeling, specifically BERTopic, has been successfully applied in various contexts, such as Twitter (subsequently rebranded X) tweets [20]. Our study extends these findings and demonstrates the potential to enhance the CM process, specifically the idea synthesis step, using various health care–related datasets. IdeaDistiller, our proposed approach, significantly reduces the manual effort traditionally required to identify and group similar ideas by automating the clustering and thereby presorting the participant-generated ideas. This automation enables researchers to allocate more time to the subsequent analysis and interpretation of ideas rather than the manual data processing phase.

Moreover, the approach provides additional outputs, including coherence scores and semantically sorted clusters, which facilitate a more structured and efficient review process. IdeaDistiller is designed to support CM researchers, while keeping them in control of their own analysis. Extracting final, unique ideas from each cluster still necessitates human judgment, and suggested representative ideas require expert validation, particularly in complex or sensitive domains such as health care.

Our findings align with Prescott et al [23], who argue that while generative artificial intelligence (AI) can significantly reduce time and resource demands, hybrid approaches incorporating HITL validation remain necessary. Similarly, Morgan [24] concludes that although AI tools demonstrate great promise for supporting coding tasks, integrating AI with qualitative data analysis presents challenges. In agreement with Prescott et al [23], human analysts retain a crucial advantage in identifying nuanced meanings and interpreting context-specific themes—an essential skill for rigorous qualitative research.

Integrating an automated step into the idea synthesis process offers substantial benefits for methods such as CM. These include improved scalability for handling larger datasets. We also concur with Chen et al [25], who advocate for using machine learning to identify inconsistencies and ambiguities in qualitative coding. By automating the simpler stages of coding, researchers can allocate their expertise more efficiently to the complex interpretative tasks that require theoretical sensitivity and contextual knowledge—an outcome facilitated by the tool developed in this study.

### Limitations

A limitation encountered in this study was the variability in dataset sizes and languages, which posed challenges for optimal parameter tuning. Smaller datasets constrained the ability of machine learning models to generalize effectively. Nevertheless, by using stable parameter settings, we achieved consistent and satisfactory performance across most datasets.

While a HITL approach remains indispensable for analyzing complex qualitative data, developing systems that support and enhance this collaboration is a promising direction for the future evolution of the CM methodology. Further application of the approach across diverse contexts will be necessary to fully understand its capabilities and identify opportunities for improvement.

In summary, our findings suggest that IdeaDistiller can significantly streamline a previously time-consuming step of the CM process, improve scalability, and maintain reasonable accuracy. Nonetheless, human validation remains essential to ensure the quality and relevance of results in real-world applications.

### Future Work

In this study, we created IdeaDistiller, a tool trained on previous CM studies. An important next step will be to use and test the tool in further real-life studies across different domains to enable continued evaluation with respect to both usability and the experience of time-saving.

Additionally, advances in explainable AI could be leveraged to improve the interpretability of clustering results by providing insights into how and why specific items are grouped together. For instance, techniques such as attention heatmaps, feature importance scores, or natural language explanations could help domain experts understand the rationale behind each cluster, thereby enhancing transparency, supporting critical evaluation, and increasing trust in the system’s outputs.

Overall, the continued development of semiautomated tools, coupled with human expertise, presents a compelling path forward for improving the efficiency, scalability, and methodological rigor of CM and other qualitative research methodologies.

## Abbreviations

AI: artificial intelligence
BERT: bidirectional encoder representations from transformers
BERTopic: bidirectional encoder representations topic modeling
CM: concept mapping
HITL: human-in-the-loop
KB-BERT: Kungliga Biblioteket bidirectional encoder representations from transformers
SVD: singular value decomposition

## References

[1] Kane M, Trochim WMK (2007). Concept Mapping for Planning and Evaluation.

[2] Pantha S, Jones M, Gartoulla P, Gray R (2023). A systematic review to inform the development of a reporting guideline for concept mapping research. Methods Protoc.

[3] Vaughn LM, Jones JR, Booth E, Burke JG (2017). Concept mapping methodology and community-engaged research: a perfect pairing. Eval Program Plann.

[4] Rosas SR, Kane M (2012). Quality and rigor of the concept mapping methodology: a pooled study analysis. Eval Program Plann.

[5] Mclinden D, Vaughn LM (2016). Handbook of Methodological Approaches to Community-Based Research: Qualitative, Quantitative, and Mixed Methods.

[6] Trochim W, Kane M (2005). Concept mapping: an introduction to structured conceptualization in health care. Int J Qual Health Care.

[7] Ashe MC, Azim FT, Ariza-Vega P (2022). Determinants of implementing reablement into research or practice: a concept mapping study. Physiother Res Int.

[8] Windsor LC (2013). Using concept mapping in community-based participatory research: a mixed methods approach. J Mix Methods Res.

[9] Hassmiller Lich K, Urban JB, Frerichs L, Dave G (2017). Extending systems thinking in planning and evaluation using group concept mapping and system dynamics to tackle complex problems. Eval Program Plann.

[10] McLinden D (2017). And then the internet happened: thoughts on the future of concept mapping. Eval Program Plann.

[11] Vaughn LM, Jacquez F, Marschner D, McLinden D (2016). See what we say: using concept mapping to visualize Latino immigrant’s strategies for health interventions. Int J Public Health.

[12] Montgomery L, Vaughn LM, Jacquez F (2022). Engaging adolescents in the fight against drug abuse and addiction: a concept mapping approach. Health Educ Behav.

[13] Vaughn LM, Sunny CE, Lindquist-Grantz R (2020). Successful suicide screening in the pediatric emergency department: youth, parent, researcher, and clinician perspectives. Arch Suicide Res.

[14] Smith F, Fredriksson K, Gunnarsdóttir KÁ, Holtenman M, Carlsson C (2025). Increasing credibility in government assignments: an example from Sweden of stakeholder involvement by using concept mapping. BMJ Open Qual.

[15] Smith F, Hellström A, Gunnarsdóttir KÁ (2021). Exploring the meaning, role and experiences of a patient-led social innovation for people affected by cancer: a new collaborative care model complementing traditional cancer rehabilitation in Sweden. BMJ Open Qual.

[16] Smith F, Gunnarsdóttir KÁ, Genell A (2019). Evaluating the implementation and use of the regional cancer plan in Western Sweden through concept mapping. Int J Qual Health Care.

[17] Jelodar H, Wang Y, Yuan C (2019). Latent Dirichlet allocation (LDA) and topic modeling: models, applications, a survey. Multimed Tools Appl.

[18] Blei DM, Ng AY, Jordan MI (2003). Latent Dirichlet allocation. J Mach Learn Res.

[19] Grootendorst M (2022). BERTopic: neural topic modeling with a class-based TF-IDF procedure. arXiv.

[20] Egger R, Yu J (2022). A topic modeling comparison between LDA, NMF, Top2Vec, and BERTopic to demystify Twitter posts. Front Sociol.

[21] Devlin J, Chang MW, Lee K, Toutanova K BERT: pre-training of deep bidirectional transformers for language understanding. https://aclanthology.org/N19-1423.pdf.

[22] Reimers N, Gurevych I Sentence-BERT: sentence embeddings using Siamese BERT-networks.

[23] Prescott MR, Yeager S, Ham L (2024). Comparing the efficacy and efficiency of human and generative AI: qualitative thematic analyses. JMIR AI.

[24] Morgan DL (2023). Exploring the use of artificial intelligence for qualitative data analysis: the case of ChatGPT. Int J Qual Methods.

[25] Chen NC, Drouhard M, Kocielnik R, Suh J, Aragon CR (2018). Using machine learning to support qualitative coding in social science: shifting the focus to ambiguity. ACM Trans Interact Intell Syst.

[26] IdeaDistiller. Chalmers GitLab.

